# Nontoxigenic Vibrio cholerae Challenge Strains for Evaluating Vaccine Efficacy and Inferring Mechanisms of Protection

**DOI:** 10.1128/mbio.00539-22

**Published:** 2022-04-07

**Authors:** Bolutife Fakoya, Karthik Hullahalli, Daniel H. F. Rubin, Deborah R. Leitner, Roma Chilengi, David A. Sack, Matthew K. Waldor

**Affiliations:** a Division of Infectious Diseases, Brigham & Women’s Hospital, Boston, Massachusetts, USA; b Department of Microbiology, Harvard Medical School, Boston, Massachusetts, USA; c Howard Hughes Medical Institute, Bethesda, Maryland, USA; d Enteric Disease and Vaccine Research Unit, Centre for Infectious Disease Research in Zambia, Lusaka, Zambia; e Department of International Health, Johns Hopkins Bloomberg School of Public Health, Baltimore, Maryland, USA; Harvard School of Public Health

**Keywords:** cholera, human challenge, vaccine testing

## Abstract

Human challenge studies are instrumental for testing cholera vaccines, but these studies use outdated strains and require inpatient facilities. Here, we created next-generation isogenic Ogawa and Inaba O1 V. cholerae challenge strains (ZChol strains) derived from a contemporary Zambian clinical isolate representative of current dominant pandemic V. cholerae. Since the primary mechanism of immune protection against cholera is thought to be antibody responses that limit V. cholerae colonization and not the diarrheagenic actions of cholera toxin, these strains were rendered nontoxigenic. In infant mice, the ZChol strains did not cause diarrhea and proved to accurately gauge reduction in intestinal colonization mediated by effective vaccination. ZChol strains were also valuable as targets for measuring vibriocidal antibody responses. Using barcoded ZChol strains, we discovered that vaccination and passive immunity in the infant mouse model tightens the infection bottleneck without restricting pathogen expansion during intestinal infection. Collectively, our findings suggest that ZChol strains have the potential to enhance the safety, relevance, and scope of future cholera vaccine challenge studies and be valuable reagents for studies of immunity to cholera.

## INTRODUCTION

Diarrheal diseases remain one of the leading causes of infectious disease death globally and cholera, caused by the human bacterial pathogen Vibrio cholerae, accounts for approximatively 100,000 deaths each year (1, 2). Cholera is endemic in over 50 countries and often spreads explosively during epidemics (1) by the fecal-oral route and/or through ingestion of contaminated food or water. V. cholerae replicate in the human small intestine (SI) and secrete cholera toxin (CT), an AB_5_ type exotoxin that causes profuse secretory diarrhea, the clinical hallmark of cholera (3). Rehydration therapy is the mainstay of cholera treatment, with antibiotics being used in severe clinical cases. Several vaccine formulations have been developed to prevent cholera, and three killed whole-cell oral vaccine preparations are WHO prequalified, two of which, Euvichol and Shanchol, are distributed through a global stockpile (4, 5). It is thought that eradicating cholera globally will require multisectoral public health approaches, including vaccines, enhanced surveillance, as well as global water, sanitation, and hygiene initiatives (6).

Though there are over 200 serogroups of V. cholerae, only serogroup O1 has given rise to cholera pandemics (3). Serogroup O1 V. cholerae is further classified into Ogawa and Inaba serotypes that differ in the methylation of the terminal perosamine of the O-antigen of V. cholerae lipopolysaccharide (LPS); Ogawa strains are methylated, and Inaba strains are unmethylated (7). Biotype is another key classifier of pandemic V. cholerae; the first six pandemics were caused by the now extinct classical biotype (8), whereas the ongoing 7th pandemic is caused by the El Tor biotype (3). Molecular epidemiology has identified 3 distinct clades (waves) of pandemic V. cholerae within the 7th pandemic that have emerged and disseminated from South Asia to other continents. Wave 3 7th pandemic El Tor (7PET) strains are responsible for the dominant circulating V. cholerae globally and have caused devastating outbreaks in Haiti, Yemen, and South Asia (9–11). Currently, sub-Saharan Africa carries the dominant burden of cholera cases reported to the WHO (12).

Both animal and human studies suggest that immune responses targeting the O-antigen of V. cholerae LPS are critical for immune protection from cholera, but responses to other antigens, including CT, may also contribute to protection. Studies of cholera patients, as well as healthy volunteers, challenged with wild-type V. cholerae revealed that infected persons develop serum and intestinal antibody responses to LPS and CT and were protected from future episodes of cholera (13–15). These observations motivated development of vaccines designed to stimulate immune responses similar to those stimulated by the disease (16) and led to the development of killed whole-cell oral vaccines (3), as well as a live attenuated oral vaccine (17). Serologic correlates of protection are often useful to predict whether disease or vaccination induces protection (18, 19). For cholera, the vibriocidal antibody titer (VAT) response following vaccination does suggest protection but is not an effective correlate of long-term immunity because VAT titers fall within a few months after vaccination, but protection lasts for several years (13, 15, 20). Thus, proof of vaccine efficacy has depended on testing of vaccines using human volunteers or in controlled field trials.

Following a controlled human infection model (CHIM) that demonstrated that a killed whole-cell oral cholera vaccine provides protection (21), large, double-blind, placebo-controlled field trials further demonstrated their efficacies in India (20) and Bangladesh (22), and subsequent case-control studies confirmed effectiveness in Africa (23). A live attenuated vaccine was licensed for travelers based on results from a CHIM study that demonstrated protection for at least 3 months (24), showcasing how CHIM studies have played a crucial role in cholera vaccine development. In these studies, the rate and severity of diarrheal illness in vaccinated and naive volunteers who orally consume virulent V. cholerae is compared (24, 25). Typically, most of the unvaccinated, naive volunteers develop diarrhea and/or vomiting for 1 to 3 days and require rehydration. Because of the risk of illness, cholera CHIMs are only carried out in specialized inpatient facilities where healthy volunteers are cared for by experienced physicians. CHIM studies for cholera were initially carried out by Cash et al. to determine V. cholerae infectious doses and evaluate vaccine efficacy (14). CHIM studies have also revealed that antibacterial immune responses lead to reductions in fecal excretion of challenge strains, which generally correlate with vaccine efficacy (25).

Currently, the V. cholerae strain used in most CHIMs is N16961, a 1971 7PET wave 1 Inaba isolate (24–27). Although N16961 has been widely used for CHIM studies, there are several compelling reasons why a new challenge strain would be valuable for cholera CHIM studies. First, N16961 is a wave 1 V. cholerae strain that differs considerably from currently circulating wave 3 V. cholerae, including in virulence-associated loci such as *tcpA* and *ctxB* (9). Second, the N16961 strain exhibits hypervirulence in animal models of infection (28). Third, since many of the volunteers develop severe diarrhea and vomiting, a CHIM strain that does not cause severe illness and without the need for a specialized center would be preferable if it provides evidence for vaccine protection. Given the potential risks to volunteers and biosafety concerns associated with toxigenic V. cholerae, cholera CHIM studies have not been carried out in regions of cholera endemicity (29, 30).

CHIMs have played a vital role in guiding cholera vaccine development, and since N16961 has significant limitations, we engineered V. cholerae strains as potential next-generation V. cholerae strains for volunteer challenge studies. These ZChol strains are derived from a 2016 7PET wave 3 clinical isolate from Zambia. To reduce the risk for volunteers and to potentially enable their conduct in countries of cholera endemicity, we rendered the strains nontoxigenic through deletion of *ctxAB* (CT). An isogenic Inaba version of ZChol was also generated, and both ZChol strains colonized the intestine of infant mice to similar levels as the wild-type toxigenic parent strain (ZTox). We show that these strains robustly report on vaccine-mediated protection both by CFU reduction and as VAT detection reagents. Finally, using ZChol strains barcoded with thousands of genomic sequence tags, we demonstrate the utility of barcoded and nontoxigenic challenge strains to decipher mechanisms of vaccine protection. Together, these results indicate that the ZChol strains will facilitate an expanded range of safer V. cholerae CHIMs as well as laboratory studies of immunity to V. cholerae.

## RESULTS

### ZTox is a wave 3 seventh pandemic El Tor V. cholerae strain.

**FIG 1:**
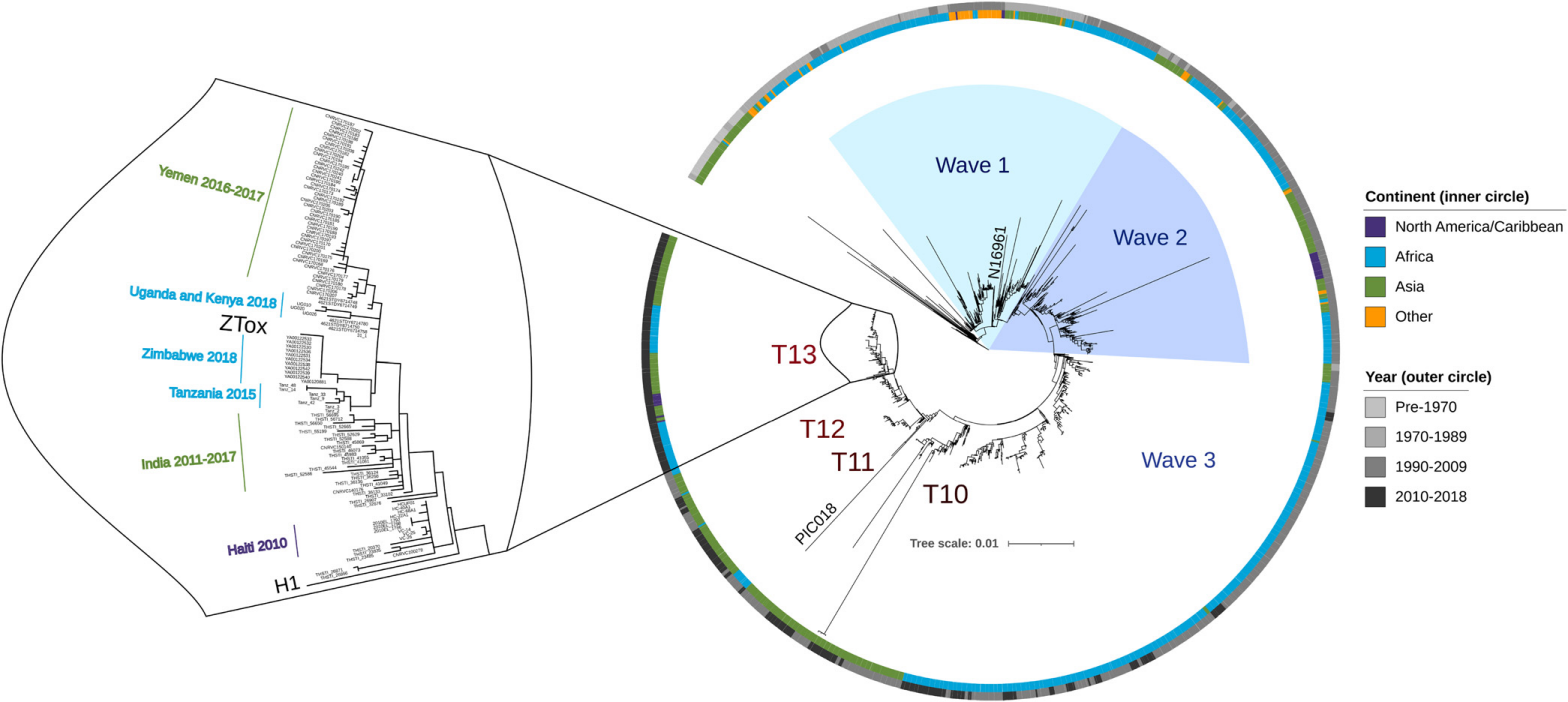
Position of ZTox in the phylogeny of pandemic V. cholerae. A maximum-likelihood phylogenetic tree from ∼1,200 toxigenic clinical isolates of V. cholerae with overrepresentation of contemporary clinical isolates from outbreaks in sub-Saharan Africa (light blue). The preseventh pandemic strain A6 (31) was used as an outgroup. Scale bar represents the mean number of nucleotide substitutions per site. ZTox clusters with 7PET wave 3 strains, particularly with isolates from nearby African countries (sublineage T13) and South Asian regions of V. cholerae endemicity that seed long-range transmission events. The PIC018 Inaba strain used for vibriocidal assays is also depicted.

The V. cholerae strain that is most frequently used in challenge studies for testing vaccine efficacy, N16961, was isolated 5 decades ago and is no longer representative of the dominant globally circulating V. cholerae. We chose a 2016 V. cholerae O1 Ogawa clinical isolate from Zambia for development as a new challenge strain, reasoning that this isolate would be representative of contemporary pandemic V. cholerae. A combination of long- and short-read next-generation sequencing (Nanopore and Illumina, respectively) was used to compile this strain’s genome and determine where it falls within a representative phylogeny of toxigenic V. cholerae that was created using ∼1,200 clinical V. cholerae isolates (31), including a substantial collection of contemporary African isolates. Comparison of ZTox with known toxigenic V. cholerae clinical isolates revealed that it clusters with 7PET wave 3 strains (Fig. 1), the dominant cause of cholera worldwide today. Genomic analyses of clinical V. cholerae isolates from Africa have revealed multiple reintroduction events of V. cholerae onto the continent via long-range transmission from South Asia (32). The most recent of these events, which gave rise to the T13 sublineage, originates from the same clade that was introduced from Nepal to Haiti and gave rise to the Haitian cholera epidemic that began in late 2010 (33). ZTox is found within this T13 sublineage and clusters with recent outbreak strains from Southern and East Africa (34). ZTox is also closely related to the V. cholerae strains that originated the devastating outbreak in Yemen throughout late 2016 (33). These analyses suggest that this isolate is typical of present-day clinically relevant wave 3 El Tor V. cholerae.

Analyses of important mobile elements within the ZTox genome also confirmed it is representative of contemporary wave 3 El Tor V. cholerae. The CTXΦ prophage in ZTox contains a single CTXΦ prophage and an adjacent RS1 satellite prophage (35). Both elements encode the El Tor CTX phage repressor (rstR^ET^) (Fig. S1A in the supplemental material). Like other wave 3 T13 strains, the ZTox CTX prophage harbors the *ctxB7* allele that has been associated with elevated secretion of cholera toxin (Fig. S1A). The SXT integrative conjugative element (ICE) commonly confers resistance to several antibiotics, including sulfamethoxazole and low levels of chloramphenicol and streptomycin, and has been linked to evolution of both wave 2 and 3 7PET V. cholerae (11). However, like many other recent African T13 strains, ZTox is sensitive to these antibiotics. Notably, the ZTox-SXT lacks *sul2*, *floR*, and *aph3* and *aph6* that confer resistance to these agents (Fig. S1B) and is nearly identical to the SXT/R391 ICE*VchInd*5 that was first described in a 2009 study characterizing a V. cholerae SXT ICE isolated from India in 1994 (36). These observations are consistent with the idea that a recent ancestor of ZTox was derived from *a* V. cholerae strain from South Asia. Other predicted genomic features of ZTox are identified in Table S2.

10.1128/mbio.00539-22.1 FIG S1 (A) Genomic organization of ZChol CTXΦ prophage as well as the RS1 satellite phage. The cholera toxin genes are indicated in red and were deleted from ZTox to generate ZChol^O^. (B) Comparison of SXT ICEs found in ZTox and 7PET H1 V. cholerae. The annotated ZTox-SXT has almost complete consensus homology with the H1-SXT ICE (green consensus bar) by nucleotide alignment with MUSCLE. Like the ICE*VchInd*5 originally described in a V. cholerae strain isolated in 1994 in India (36), the ZTox-SXT lacks a 12.49-kb region that codes for multiple antibiotic resistance genes as well as two previously unannotated predicted proteins. The borders of the missing region are flanked by predicted transposases. Download FIG S1, PDF file, 0.2 MB. Copyright © 2022 Fakoya et al. 2022 Fakoya et al. https://creativecommons.org/licenses/by/4.0/ This content is distributed under the terms of the Creative Commons Attribution 4.0 International license.

10.1128/mbio.00539-22.4 TABLE S2 Predicted gene contents of V. cholerae pathogenicity islands using the Vicpred (52) analysis pipeline. Download Table S2, PDF file, 0.2 MB. Copyright © 2022 Fakoya et al. 2022 Fakoya et al. https://creativecommons.org/licenses/by/4.0/ This content is distributed under the terms of the Creative Commons Attribution 4.0 International license.

### Generation of isogenic nontoxigenic Ogawa and Inaba derivatives of ZTox.

We reasoned that deletion of *ctxAB* (CT) could potentially expand the utility of ZTox derivatives for use both as a challenge strain and as targets for vibriocidal assays by removing the major diarrhoeagenic component, CT, of V. cholerae. The resulting strain, ZChol^O^ (Ogawa), had growth kinetics in standard laboratory conditions (LB media) that were indistinguishable from ZTox (Fig. S2A) and, as expected, did not produce CT when grown in laboratory (AKI) conditions that induce CT synthesis in V. cholerae (Fig. S2B). The *wbeT* gene encoding the perosamine methyltransferase was deleted from ZChol^O^ to yield ZChol^I^, an Inaba version of this nontoxigenic strain (Fig. S2C). We anticipate that the nontoxigenic isogenic ZChol^O^ ZChol^I^ pair should be valuable to gauge serotype-specific responses to cholera and cholera vaccines. Both strains are susceptible to antibiotics such as azithromycin, tetracycline, combinations of sulfamethoxazole and trimethoprim, and ciprofloxacin (Table S1) that are often used to treat cholera; these agents could be used if these ZChol strains prove diarrheagenic in human studies.

10.1128/mbio.00539-22.2 FIG S2 (A) Neither the *rpsL* K88R streptomycin resistance mutation nor deletion of *ctxAB* influences the growth rate of ZChol^O^ strains under standard laboratory conditions. (B) Western blot analysis of CT production under standard laboratory growth conditions as well as AKI virulence gene induction conditions. Immunoblots were loaded with equal amounts of sterile-filtered supernatants of AKI-grown ZTox (lane 1) and ZChol^O^ (lane 2) as well as with sterile-filtered supernatants of LB-grown ZTox (lane 3) and ZChol^O^ (lane 4) and incubated with an anti-CT polyclonal antibody (Abcam; catalog no. ab123129). The sizes of the A and A1 subunit (A and A1) as well as the B subunit (B) of CT are indicated by arrows on the right. Lines to the left indicate the molecular masses of the protein standards in kDa. (C) Slide agglutination analysis using serotype specific antiserum demonstrates that ZChol^O^ and ZChol^I^ are Ogawa and Inaba serotypes, respectively. (D) Barcoded ZTox and ZChol strains were serially diluted and plated for CFU and then enumerated. Sequencing of these libraries showed that the Ns/CFU relationship correlated well and that Ns is a good predictor of known CFU values. Download FIG S2, PDF file, 0.1 MB. Copyright © 2022 Fakoya et al. 2022 Fakoya et al. https://creativecommons.org/licenses/by/4.0/ This content is distributed under the terms of the Creative Commons Attribution 4.0 International license.

10.1128/mbio.00539-22.3 TABLE S1 Both ZChol^O^ and ZChol^I^ are sensitive to antibiotics commonly used to treat V. cholerae. S, sensitive; R, resistant; N, indeterminate. Download Table S1, PDF file, 0.08 MB. Copyright © 2022 Fakoya et al. 2022 Fakoya et al. https://creativecommons.org/licenses/by/4.0/ This content is distributed under the terms of the Creative Commons Attribution 4.0 International license.

### ZChol^O^ and ZChol^I^ colonize the infant mouse intestine but do not cause morbidity.

**FIG 2:**
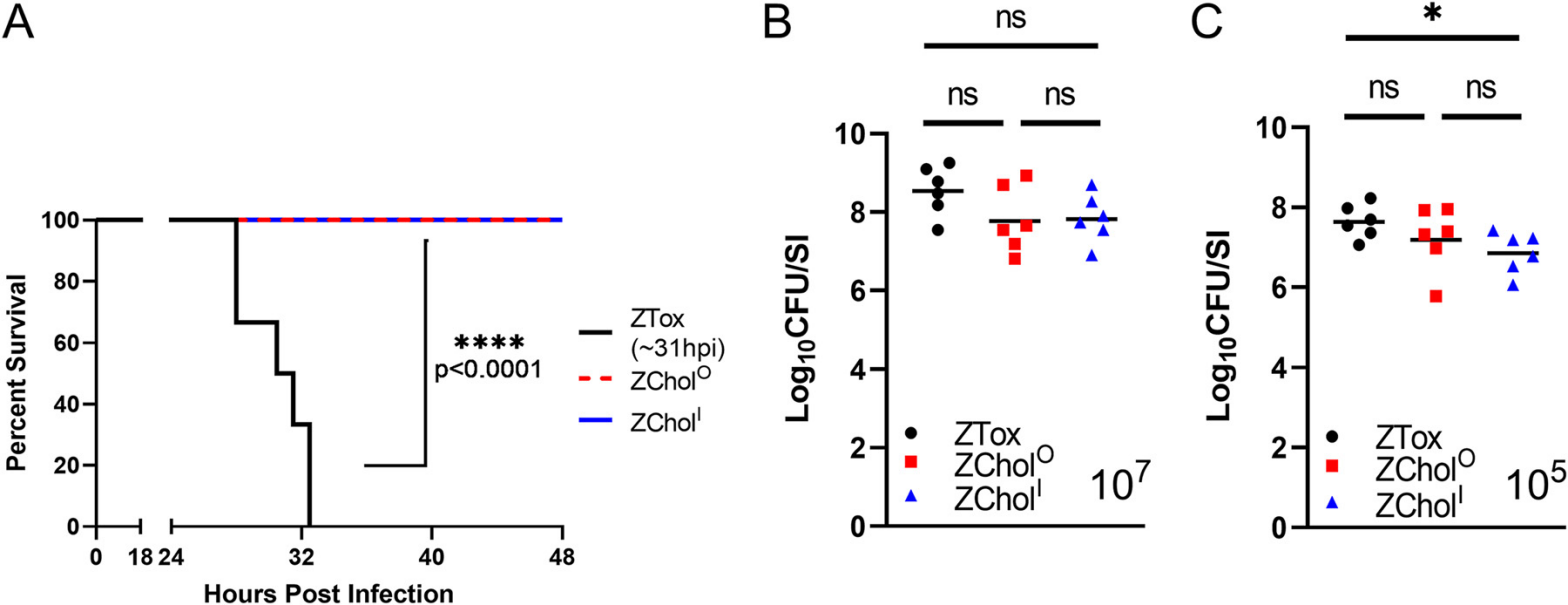
ZChol^O^ and ZChol^I^ robustly colonize the small intestine but do not cause death. (A) Kaplan-Meier plot of P3 infant mice orally infected with a lethal dose of 10^7^ CFU of the indicated strains; *n* = 6 per group. Median time to moribundity was 31 hours postinfection for infant mice infected with the toxigenic parent strain. Differences in the survival curves were assessed with a log-rank (Mantel Cox) test. (B) V. cholerae burden in the small intestine (SI) of challenged mice at time of moribundity (ZTox) or at 48 hours postinfection (ZChol strains). (C) CFU per SI for P5 infant mice orally infected with a nonlethal 10^5^ CFU of indicated strains and sacrificed at 20 hours postinoculation. Differences in CFU burdens were assessed with a Mann-Whitney U test. Horizontal bars indicate geometric means of each group. ns, not significant, * *p* < 0.05.

We used the infant mouse model of cholera to compare the lethality as well as the intestinal colonization of ZChol^O^ and ZChol^I^ with the toxigenic ZTox parent strain. In this model, diarrhea and morbidity are dependent on CT activity (37). A 10^7^ CFU oral dose routinely leads to diarrheal disease and death/moribundity within 48 hours with toxigenic V. cholerae strains (28, 37, 38). All pups given ZTox developed diarrhea and became moribund by 35 h postinoculation with a median time to death of 31 hours across 3 independent litters (Fig. 2A). In marked contrast, none of the animals inoculated with ZChol strains developed diarrhea or illness, and all 6/6 and 6/6 of ZChol^O^ and ZChol^I,^ respectively, were alive at 48h post inocuation (Fig. 2A). Small intestinal CFU burdens were determined when animals became moribund (in the case of ZTox) or at 48 hours for ZChol strains. Despite the pronounced differences in the morbidity elicited by these strains, they all colonized the small intestine to a similar extent (Fig. 2B).

In a complementary experiment, a lower dose of 10^5^ CFU was used to directly compare the capacities of ZTox and ZChol^O^ and ZChol^I^ to colonize the SI at 20 hours postinoculation, a point where less diarrheal disease was apparent. The small intestinal CFU burdens in infected mice were also similar using this protocol, though the ZChol^I^ burden was modestly lower than that of ZTox (Fig. 2C). Together, these data suggest that the engineered ZChol strains pose reduced risk of inducing cholera-like disease in human challenge studies but, nevertheless, would be useful indicators of V. cholerae intestinal colonization.

### Utility of ZChol strains as challenge strains for vaccine studies.

**FIG 3:**
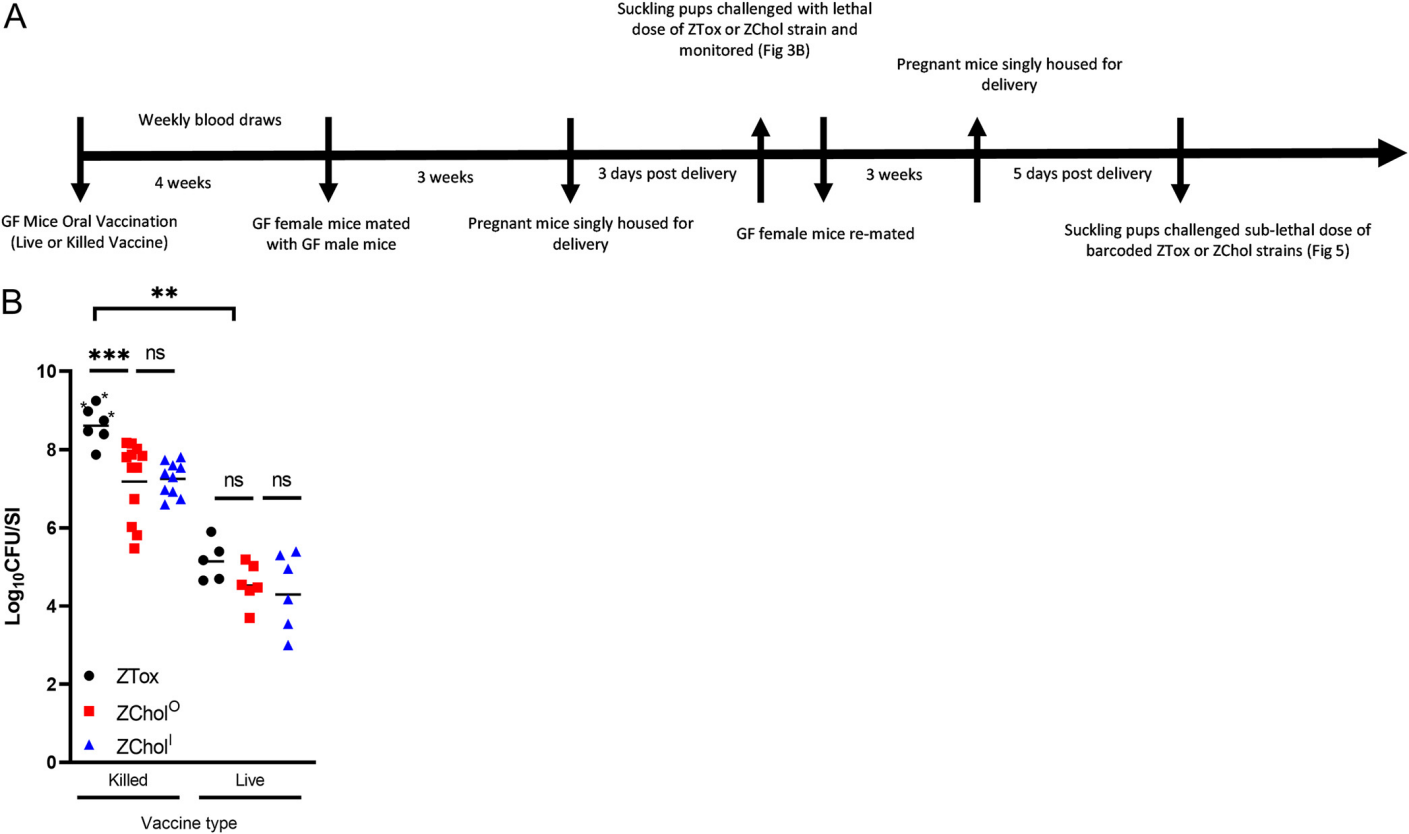
ZChol^O^ and ZChol^I^ provide a similar gauge of vaccine-mediated protection as ZTox without causing diarrheal disease. (A) Schematic overview of GF vaccination regimen and experimental protocol. (B) P5 pups born to germfree female mice orally immunized with either a live (*n* = 5 GF dams) or killed oral cholera vaccine (OCV) (*n* = 5 GF dams) were orally inoculated with ZTox or ZChol^O^ or ZChol^I^. CFU per SI in these pups were determined at 20 hours post inoculation (10^5^ CFU per infant mouse inoculum) with the indicated strains. Asterisks denote mice with visible diarrhea. Differences in CFU burdens were determined by a Mann-Whitney U test. Horizontal bars indicate geometric means of each group.

To model how ZChol strains could be used in human challenge studies, we measured how colonization with these strains could be used to determine vaccine efficacy (Fig. 3A). We immunized adult germfree (GF) mice with killed and live formulations of the PanChol oral cholera vaccine (OCV) (28, 39), a highly engineered live attenuated OCV derived from the V. cholerae strain that caused the 2010 cholera outbreak in Haiti, and then challenged the pups of the immunized dams with either ZTox, ZChol^O^, or ZChol^I^ (Fig. 3A). In the GF model, live oral cholera vaccines are far more potent immunogens than killed whole-cell cholera vaccines, which have minimal efficacy in this model as single-dose agents (28). In this model, the colonized dams do not transmit PanChol to their pups; protection is passive due to the transfer of antibodies in milk to the nursing pups (37). Half of the pups from dams immunized with the killed vaccine exhibited diarrhea and inactivity upon ZTox challenge, whereas none of the 34 mice challenged with ZChol^O^ or ZChol^I^ developed signs of disease (Fig. 3B). Independent of challenge strain, the neonatal mice born to and suckled by dams that had received the live vaccine had significantly lower SI V. cholerae burdens than the mice from the killed vaccine group. This observation reflects the greater potency of the live vaccine and suggests that ZChol colonization can be used to gauge vaccine protection while reducing the risk inherent in challenge with toxigenic V. cholerae. In the killed PanChol vaccine group, the intestinal burden of ZChol strains was modestly lower in animals challenged with these nontoxigenic strains than ZTox (Fig. 3B). However, there were no significant differences in intestinal burdens of ZTox, ZChol^O^, or ZChol^I^ in animals in the live vaccine group. Together, these data demonstrate the potential for ZChol strains to be used in derisked human CHIM studies.

### ZChol strains are advantageous reagents for measuring vibriocidal antibody titers.

The closest correlate of protective immunity against cholera is circulating vibriocidal antibody titers (VATs). Vibriocidal assays use Ogawa and Inaba strains, such as PIC158 and PIC018, respectively (40), which are toxigenic and nonisogenic, as targets, along with exogenous complement, to measure the titer of complement fixing anti-Ogawa and anti-Inaba antibodies in serum samples. PIC018 was isolated from Bangladesh in 2007 and is relatively genetically distant from many currently circulating strains (Fig 1); using a more contemporary target strain may allow for greater sensitivity to contemporary V. cholerae antigens. We reasoned that ZChol strains, which are genetically identical except for the *wbeT* deletion, could be useful as target strains for measurements of serotype-specific vibriocidal antibodies because they only differ in the methylation of the V. cholerae O1 O-antigen and not in other potential targets of vibriocidal antibodies. Furthermore, these strains pose a lower biosafety risk to laboratory workers because they lack the CT genes.

**FIG 4:**
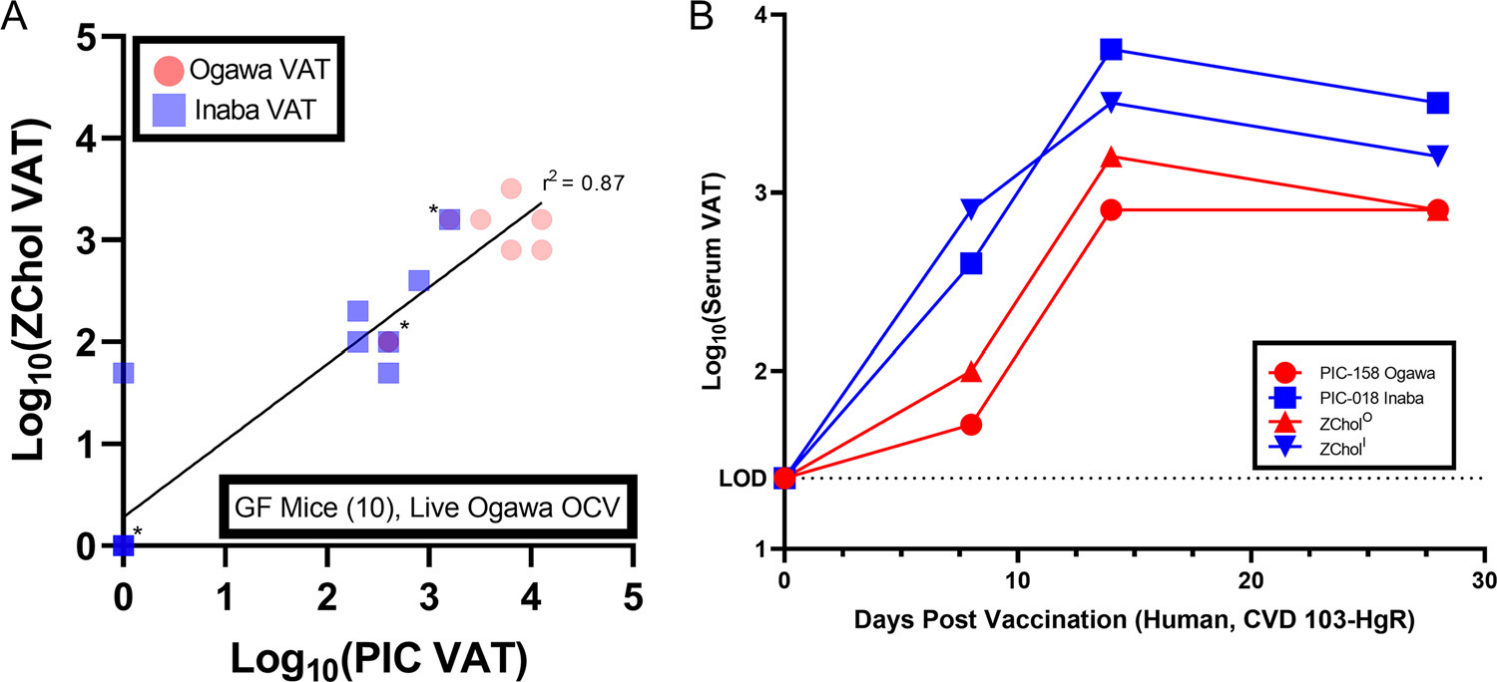
Measurement of vibriocidal antibody titers in both murine and human convalescent-phase serum using ZChol^O^ or ZChol^I^ as targets. (A) ZChol strains were used to determine the vibriocidal antibody titers (VATs) of live Ogawa OCV-immunized GF mouse serum (*n* = 10) from a previous study (28) and compared to previously reported VAT values from nonisogenic PIC reporter strains. Red circles represent VATs determined by ZChol^O^ and PIC-158 (Ogawa) reporter strains, and the blue squares represent VATs determined by ZChol^I^ and PIC-018 (Inaba) reporter strains. VATs below the limit of detection were set to 1 for statistical analysis. The *r*^2^ linear correlation value between the PIC and ZChol strains was 0.87. Asterisks denote overlapping data points. (B) ZChol strains and PIC strains were both used to determine the VATs from human serum obtained pre- and postvaccination with CVD 103-HgR (Inaba live OCV). Dashed line represents the limit of detection (LOD) of this assay.

We tested the ZChol pair as target strains in vibriocidal assays using murine serum obtained from a previous cholera vaccine study (28), with the identical protocol used to measure serotype-specific VATs in the previous study where PIC018 and PIC158 were used as the target strains. The correlation (*r*^2^ value) between the VATs reported using the PIC strains and those determined using the ZChol strains was 0.87 (Fig. 4A). In one sample, there was a detectable anti-Inaba VAT using ZChol^I^ that was not detected by the Inaba PIC018 strain (Fig. 4A), suggesting that ZChol^I^ may be a more sensitive indicator strain than PIC018.

Similar experiments were carried out to quantify VATs from serum obtained from a human volunteer vaccinated with CVD103-HgR, a live attenuated Inaba oral cholera vaccine (OCV). Again, ZChol strains PIC158 and PIC018 were used as targets in parallel VAT assays (Fig. 4B). Both sets of reporter strains revealed the kinetics of the vibriocidal responses to a single dose of this live attenuated vaccine, as well as the greater titer of anti-Inaba responses, which peaked 14 days postvaccination (Fig. 4B). Though these serum samples are from a single vaccinated individual, the data are concordant with previously published VAT data and support the utility of the ZChol strains (19). We conclude that the ZChol matched strains are useful reagents for safely and accurately evaluating protection against cholera in this most widely used clinical assay.

### Barcoded challenge strains enable new insights into the mechanisms of vaccine protection.

**FIG 5:**
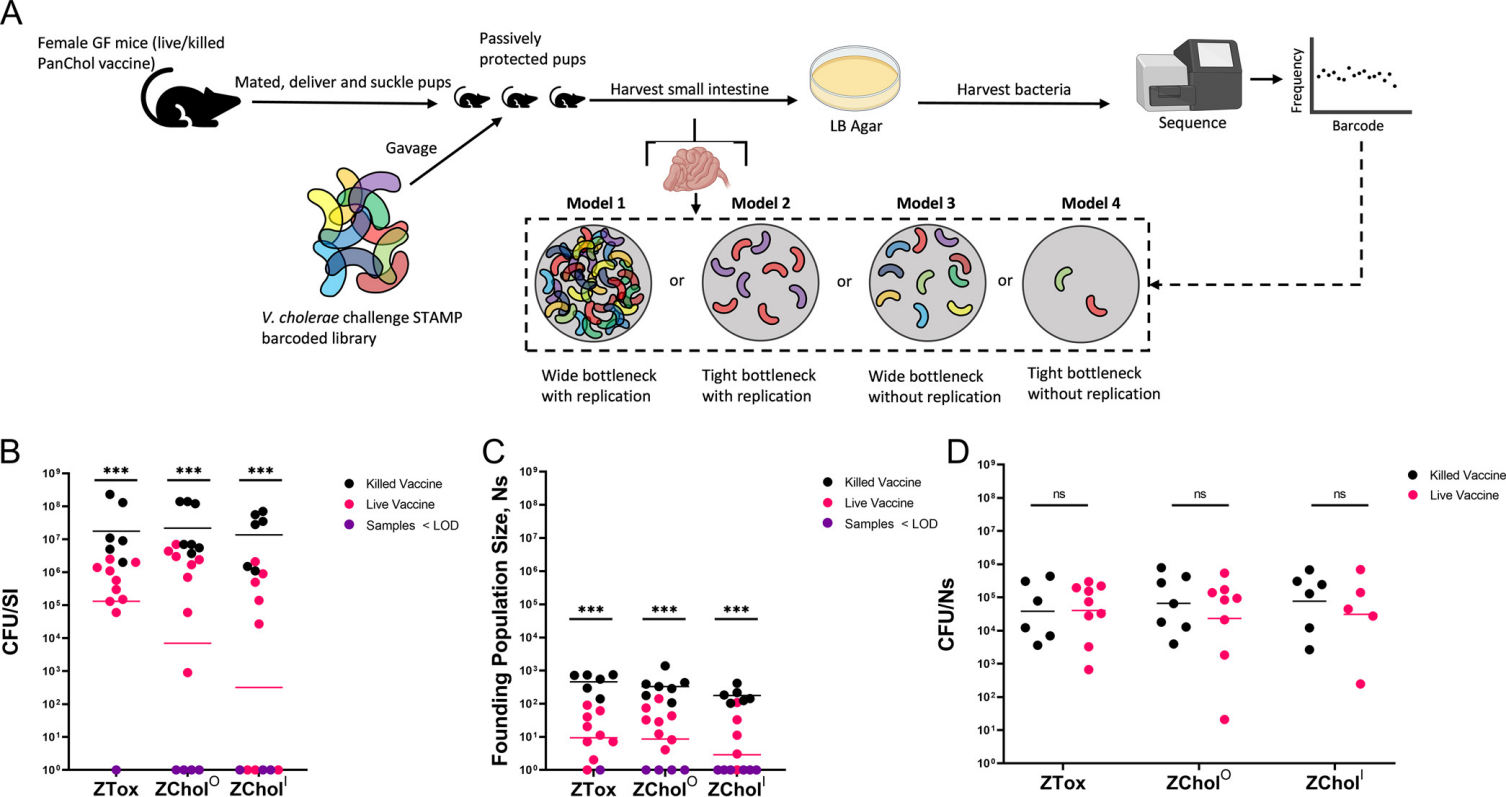
Barcoded ZChol strains can reveal the impact of vaccination on V. cholerae population dynamics during infection. (A) Schematic of experimental protocol as well as interpretations of the potential outcomes of the barcode-based analyses of bacterial population composition. Infant mice from live or killed OCV-vaccinated dams were challenged with genetically barcoded ZTox or ZChol^O^ or ZChol^I^ at a 10^5^ dose. Mice were sacrificed 20 hours postinoculation. (B) CFU per SI from each mouse orally inoculated with the barcoded libraries. Purple circles are CFU per SI values below the limit of detection of this assay, i.e., no recoverable CFU from the inoculated infant mice. (C) Ns values indicate the size of the V. cholerae founding populations, i.e., the number of bacteria from the 10^5^ CFU inoculum that successfully initiated colonization and expansion. Purple circles are Ns values below the limit of detection from deep sequencing. (D) CFU/Ns values determined by the dividing the values in panel B by the values in panel C. Purple circles are CFU/Ns imputed from values below the limit of detection. Statistical differences were determined by Mann-Whitney U tests. Color-coded horizontal bars indicate geometric means of each group. Figure was generated using www.biorender.com.

Reflecting the greater potency of live OCVs, mouse pups nursed by GF dams vaccinated with the live OCV had much lower intestinal V. cholerae burdens than those vaccinated with the killed OCV (28). Two mechanisms could account for the greater passive protection (presumably mediated by maternal antibodies) (37) observed in the live OCV group. This reduction in V. cholerae burden could be explained by antibodies restricting the number of bacteria capable of initiating colonization (the founding population) and/or by impeding bacterial expansion, which represents bacterial replication minus death and loss through defecation. We introduced ∼60,000 genomic barcodes into the genomes of ZTox, ZChol^O^, and ZChol^I^ and used our STAMPR analytic framework to distinguish between these possibilities (41). STAMPR quantifies the founding population (FP) in a metric, Ns, that reflects the number of unique cells from an inoculum that give rise to observed population in the host at the time of sampling. If passive immunity reduces bacterial expansion in the suckling pups, we expect Ns to remain unchanged while overall bacterial burden decreases. In contrast, if vaccination tightens host bottlenecks (thereby reducing the founding population size), we expect a decrease in Ns of the same magnitude as the decrease in CFU (Fig. 5A, model 2). If vaccination reduces both the founding population and bacterial expansion, we expect a decrease in Ns that is smaller in magnitude than the decrease in CFU (Fig. 5A, model 4).

As a control, Ns was first calculated across a wide range of known artificially created bottlenecks (Fig. S2D), confirming that this metric accurately quantifies bottlenecks for these libraries across 5 orders of magnitude. Then, P5 (day 5 postbirth) suckling infant mice from GF dams vaccinated with either the killed or live OCV as above were orally inoculated with the barcoded libraries of ZTox, ZChol^O^, or ZChol^I^ (Fig. 5A). As observed above (Fig. 3B), the burden of V. cholerae in pups nursed by dams vaccinated with the live vaccine was lower than those nursed by dams vaccinated with the killed OCV (Fig. 5B). The lower magnitude of difference in CFU per SI between the live and killed OCV groups (∼1.5-log_10_ fold reduction) than those observed above (Fig. 3B) is consistent with waning immunity in the vaccinated dams. Independent of the challenge strain, the live vaccine uniformly restricted the Ns values detected compared to the killed PanChol OCV (Fig. 5C). These data are consistent with the model (Fig. 5A, model 2) that passively transferred antibodies elicited by OCVs restrict the pathogen’s bottleneck but have little impact on bacterial expansion in the host. Indeed, there is no significant difference in the CFU/Ns ratio (a gauge of expansion) between pups nursed by live PanChol OCV-vaccinated or killed PanChol OCV-vaccinated animals (Fig. 5D). Together, these findings reveal that antibodies elicited by vaccination almost exclusively influence host bottlenecks and suggest that maternal antibodies within milk limit the number of bacterial cells capable of initiating infection in the suckled infant mice. However, these antibodies appear to have little influence on the capacity of the V. cholerae cells that have passed through the bottleneck and reached suitable intestinal niches to expand. Thus, vaccination in this model creates a more restrictive bottleneck without impacting the expansion of the founding population.

## DISCUSSION

Knowledge of V. cholerae pathogenesis and immunity has greatly benefitted from controlled human infection studies (CHIMs), and CHIMs using toxigenic V. cholerae challenge strains have been instrumental for testing the protective efficacy of several cholera vaccine formulations. Here, we created ZChol^O^ and ZChol^I^, isogenic Ogawa and Inaba strains, respectively, as potential next-generation V. cholerae challenge strains. These ZChol strains, which are derived from a contemporary V. cholerae isolate from Zambia, were rendered nontoxigenic to ameliorate safety concerns arising from CT-induced diarrhea. Potentially, these nontoxigenic strains might be used in outpatient studies, including in countries of endemicity, without the need for special facilities as are needed with wild-type virulent challenge strains. Finally, thousands of barcodes were introduced into a neutral genetic locus in these strains, adding the potential for analyses of V. cholerae population dynamics to CHIM studies. Our preclinical analyses presented here suggest that these strains have the potential to enhance the safety, relevance, and scope of future CHIM studies.

We chose a recent Zambian V. cholerae isolate to create the ZChol Ogawa and Inaba pair because the highest burden of reported cholera cases is now in sub-Saharan Africa. The strains have a set of genomic features that distinguish them from wave 1 strains such as N16961, which is commonly used for CHIM studies. Genomic analyses (Fig. 1) show that ZTox is representative of modern pandemic cholera, including the strains that have given rise to extensive cholera outbreaks in the past 10 to 12 years in Haiti, Yemen, and multiple African countries (32–34).

Immune responses to CT are not the principal target of long-lasting protective immunity to cholera (42). Instead, antibody responses targeting the O1 O-antigen strongly correlate with protective immunity to cholera. Such antibodies, which can be elicited by infection or cholera vaccines, prevent V. cholerae colonization and lead to marked reductions in V. cholerae shedding in CHIM studies of immunity to cholera (24). Given the reductions in V. cholerae shedding in stool in the setting of protective immunity, we reasoned that creation of nontoxigenic challenge strains may create a path to markedly derisk future CHIM challenge studies. In the infant mouse model, the ZChol strains colonized the small intestine at comparable levels to ZTox but, in contrast to the latter strain, were well tolerated (Fig. 2B). Furthermore, using a GF mouse model of vaccination and infant mouse challenge, we found that challenge with ZChol strains provided a similar measure of vaccine-elicited reduction of colonization of the challenge strain to ZTox (Fig. 3B). Since deletion of *ctxAB* does not always eliminate the diarrheagenicity of V. cholerae strains (43), it will be important to test whether the ZChol strains are minimally reactogenic prior to their adoption for human challenge studies.

While successful vaccination reduces the capacity of V. cholerae to colonize the intestine, the mechanisms that account for such reductions are not well understood. We hypothesized that vaccine-associated reductions in V. cholerae colonization could be explained by immune response-mediated narrowing of the host bottleneck(s) to infection, limiting the size of the V. cholerae-founding population, and/or by immune responses blocking intestinal V. cholerae expansion (Fig. 5A, models). To distinguish between these possibilities, ZChol strains were barcoded with ∼60,000 genomic sequence tags, and we used STAMPR (41) to quantify and compare the sizes of ZChol founding populations (FPs) in vaccinated animal models. Surprisingly, vaccination in the GF mouse model, which reflects passive immunity, appeared to only narrow the host bottleneck to infection, reducing the number of successful V. cholerae founders but did little to alter the ability of these founders to expand in the intestine. Thus, vaccine-elicited protective antibodies, which are thought to account for protection in this model, appear to largely limit the ability of V. cholerae to occupy its replicative niche in the small intestine. It will be highly informative to carry out similar experiments in the volunteer CHIM setting.

We propose that ZChol strains have the potential to dramatically alter the landscape of cholera CHIM studies and enable outpatient vaccine challenge studies in countries of cholera endemicity. Such studies could address pressing translational questions in the appropriate epidemiological settings, including optimization of dosing regimens for killed whole-cell vaccines like Shancol and Euvichol (44) and testing of new cholera vaccine candidates such as HillChol (45), PanChol (28), and conjugate vaccines (46), at far lower costs in critical settings in Africa and Asia. Beyond optimization of cholera vaccines, these new barcoded nontoxigenic strains should be valuable for studies of V. cholerae population dynamics in vaccinated and unvaccinated people across a broad set of circumstances, including studies of infectious doses in diverse settings where recipient nutritional status, prior exposure to the pathogen, and microbiota might influence mechanisms of immunity (47–49). Finally, ZChol strains should also be valuable as safe and otherwise isogenic target strains for vibriocidal and immunologic assays of the host response to cholera.

## MATERIALS AND METHODS

### Strains and growth conditions.

A V. cholerae O1 2016 clinical isolate from Zambia (EDVRU/ZM/2016), named here ZTox, was serotyped by slide agglutination using Ogawa- and Inaba-specific antisera (BD Difco). Unless otherwise indicated, all V. cholerae and E. coli strains were grown in LB medium and stored in 25% glycerol (vol/vol) at −80°C. For optimal induction of CT, V. cholerae was grown in AKI broth (0.5% sodium chloride, 0.3% sodium bicarbonate, 0.4% yeast extract, and 1.5% Bacto peptone) for 4 h without shaking, followed by 4 h with aeration at 37°C (50, 51).

### Whole-genome sequencing and phylogenetic analysis.

Genomic DNA was extracted from ZTox with the GeneJet genomic DNA purification kit (Thermo Fisher) and prepared for whole-genome sequencing. DNA for short reads was prepared using the Illumina Nextera kit (Illumina) and sequenced using a NextSeq 550 (Illumina) at the Microbial Genome Sequencing Center (MIGS; Pittsburgh, PA). Long reads were generated using the Nanopore platform (Oxford) at MIGS. Adapter trimming and quality control standards were performed with bcl2fastq, and a hybrid genome assembly was generated with Unicycler. The closed genome was then annotated using Prokka (MIGS). Raw fasta files were uploaded to Vicpred for further analyses (52). The genomic sequencing data for the strains listed in this study can be found at SRA, Bioproject ID number: PRJNA789536. Strains are available upon request to the corresponding author and are being submitted to a global repository.

Raw sequence files and assembled genomes were analyzed as in reference 31. Briefly, raw sequence files were downloaded from the European Nucleotide Archive, while 100-bp overlapping simulated reads were generated from the assembled genomes by using fasta_to_fastq.pl (https://github.com/ekg/fasta-to-fastq/blob/master/fasta_to_fastq.pl) or wgsim (https://github.com/lh3/wgsim). Simulated paired-end reads and paired-end reads were then mapped to the reference genome of 7PET wave 1 isolate N16961 (GenBank accession nos. LT907989 and LT907990) with Snippy v4.6.0 (https://github.com/tseemann/snippy) using freebayes v1.3.5 (https://github.com/freebayes/freebayes) with requirements for a mapping quality of 60, minimum base quality of 13, minimum read coverage of 4, and a 75% read concordance at a locus required for reporting. Alignments from reference 31 were kindly provided by D. Domman (University of New Mexico). The recombinogenic VSP-II region, as well as the repetitive TLC-RS1-CTX region, were then masked from the alignment using Geneious (Biomatters). Further recombinogenic sites were masked using Gubbins v3.1.2 (53). A maximum-likelihood phylogenetic tree was generated from ∼10,800 genomic single nucleotide polymorphisms (SNPs) using RAxML v8.2.12 (54) with the general time-reversible (GTR) model and 100 bootstraps. The tree was rooted on the preseventh pandemic A6 genome and visualized in iTOL v5 (55).

The SXT flanking sequence ATCATCTCGCACCCTGA was chosen from a previous comparative SXT ICE study (56) and identified in the ZTox genome. The other flank of the SXT region was then defined as the 5′ end of the *prfC* gene (57). The resulting ZTox-SXT was identified and aligned to the H1-SXT (58) in Geneious (Biomatters) using MUSCLE alignment.

### Creation of ZChol^O^ and ZChol^I^ strains.

A streptomycin-resistant variant of ZTox was isolated by plating a concentrated overnight ZTox culture onto streptomycin (Sm) LB agar plates (1 ng/mL) and grown for 24 hours at 30°C. A streptomycin-resistant (Sm^r^) colony was picked, grown in LB Sm (200 μg/mL), and frozen as a glycerol stock. Whole-genome sequencing identified the resistance mutation as *rpsL* A263G (K88R), a common allele conferring resistance to Sm (59).

A nontoxigenic (*ΔctxAB*) derivative of ZTox, ZChol^O^, was generated by allelic exchange. Initially, pBF31, a derivative of the allele exchange vector pCVD442 (carbenicillin resistance [Carb]) containing the kanamycin (Kan) resistance cassette from pKD4 (60) flanked by FLP recombination target (FRT) sites sandwiched by 1-kb homology arms targeting the *ctxAB* operon was created. BF31 was conjugated from SM10λpir Escherichia coli into Sm^r^ ZTox, and single-crossover mutants were selected on LB plus 200-μg/mL Sm (Sm200), 50-μg/mL Carb (Carb50), and 50-μg/mL Kan (Kan50) agar plates. Colonies were then grown in liquid LB plus Carb50-Kan50 at 37°C for 6 hours and then inoculated into static LB with 10% sucrose for 24 hours at room temperature. Sucrose-resistant (sacB-negative), Carb^s^, and Kan^r^ colonies were isolated and checked by colony PCR using internal and flanking primers to demonstrate the loss of *ctxAB* and the insertion of the FRT-flanked kanamycin resistance cassette. The FRT-flanked Kan^r^ cassette was removed by introducing pCP20 (60), which encodes the Flp recombinase. To accomplish this, a single colony of ZToxΔ*ctxAB*-FRT-Kan^r^ was grown in 50 mL LB with Sm200 with Kan50 μg/mL overnight at 37°C with shaking at 220 rpm and then pelleted. Pellets were washed 2× with ice-cold water and then 2× with ice-cold 10% glycerol to render them electrocompetent. Fifty microliters of electrocompetent ZToxΔ*ctxAB*-FRT-Kan^r^ was electrotransformed with 50 ng of pCP20, recovered in 1 mL (SOB) for 3 hours, and then plated on LB agar with Sm200 and Carb50 and grown overnight at 30°C. Individual Sm^r^ Carb^r^ colonies were inoculated into LB and grown at 42°C for 10 hours and then serially diluted onto LB agar with Sm200 overnight at 37°C. Individual colonies were replica struck onto LB agar with Sm200; LB Sm200 and Kan50 agar; and LB Sm200, Kan50, and Carb50 agar. Sm^r^ Kan^s^ Carb^s^ colonies were tested by colony PCR to verify the loss of the FRT-Kan^r^ yielding ZTox*ΔctxAB* (ZChol^O^). The growth of ZTox and ZChol^O^ in LB was compared in a 96-well plate format, and optical density at 600 nm (OD_600_) was determined by a plate reader to generate growth curves.

ZChol^O^ was mated with SM10λpir E. coli carrying pBF32 (pCVD442-WbeT), which contains 1-kb homology arms targeting the *wbeT* locus, and allelic exchange was carried out as described above using pCVD442 and *sacB* counterselection to isolate *ΔwbeT* strains. Colony PCR was used to confirm the resultant strain was ZChol^O^ Δ*wbeT* (ZChol^I^). Short-read whole-genome sequencing (Illumina; MIGS) was performed on both ZChol strains to verify the intended mutations. Slide agglutination with V. cholerae serotype-specific antisera (BD Difco) was performed using glass slides to confirm serotypes. Antibiotic susceptibility assays were performed using Etest strips (BioMérieux) and the results interpreted using the CLSI M45-A3 guidelines (61).

### Western blot analysis.

Proteins were separated by SDS-PAGE using 4 to 12% NuPAGE Bis-Tris precast gels (Life Technologies) and transferred to nitrocellulose using an iBlot gel transfer device (Life Technologies). The prestained protein marker, SeeBlue (Invitrogen) was used as a molecular mass standard. Rabbit anti-CT polyclonal antibody (Abcam; catalog no. ab123129) and horseradish peroxidase (HRP)-linked anti-rabbit IgG were used as primary and secondary antibodies, respectively. Blots were developed with the SuperSignal West Pico Plus chemiluminescence substrate (Thermo Fisher) and exposed in a ChemiDoc system (Bio-Rad Laboratories).

### Infant mouse lethal dose challenge assay.

Infant mouse lethal dose challenges were performed as previously described (37, 38). Five female C57BL/6 dams with 2- to 3-day-old litters (P2 to P3) (Charles River Laboratories) were singly housed with their pups. At P3 to P4, the pups within each litter were randomly assigned to three groups, orally inoculated with 10^7^ CFU in 50 μL LB of the indicated V. cholerae strain, and returned to their dams for maternal care. Inocula were serially diluted and plated in triplicate for CFU enumeration to confirm infection dose. Infected pups were closely monitored every 2 to 4 hours for the onset of diarrhea and diminished activity levels. At this point, monitoring was increased to 30-minute intervals until pups reached moribundity. When pups became moribund or at the 48-hour time point, pups were euthanized for necropsy, SI weighing, homogenization, and plating on LB Sm200 agar to enumerate the CFU per SI of the challenged mice as described previously (28, 37).

### Infant mouse colonization assay.

Three litters of 4- to 5-day-old C57BL/6 infant mice (Charles River Laboratories) were separated from their dams upon receipt and placed into isolation incubators with padding and nesting material. Inocula were prepared by making 1:1,000 dilutions of 37°C overnight LB cultures of the indicated strains (roughly 10^5^ CFU/50 μL inocula). The infant mice were randomly split into three groups, and each mouse was orally inoculated with the indicated strain and returned to the incubation chambers for 20 hours. After 20 hours, the mice were euthanized and necropsied, and CFU/SI were enumerated as described above.

### Germfree immunization study.

Ten 4-week-old C57BL/6 female germfree (GF) mice (Massachusetts Host-Microbiome Center) were maintained in a biosafety level 2 (BSL2) facility with autoclave-sterilized cages, food, and water. On day 0 of the experiment, half (five) of the mice were anesthetized and orally inoculated with 10^9^ CFU of an overnight culture of live PanChol oral cholera vaccine (OCV) in 100 μL sodium bicarbonate (28). The other half of the mice were anesthetized and given the same dose (10^9^ CFU) of formalin-killed PanChol in sodium bicarbonate (Fig. 3A). Mice were cohoused according to their vaccine group. Four weeks postvaccination, the female mice were mated with age-matched GF C57BL/6 male mice and monitored closely for signs of pregnancy. Pregnant dams were singly housed in sterilized cages beginning at ∼E-18 (3 days before delivery) for delivery. Randomly assigned suckling P3 pups from each litter were challenged with typically lethal doses (10^7^ CFU) of the indicated strains as described and returned to their respective dams for care. CFU per SI enumeration after 20 hours of infection of the pups was carried out as previously described.

### Vibriocidal antibody titers assays.

Murine and human vibriocidal antibodies were quantified by finding the highest serum dilution required to lyse isogenic ZChol strains or PIC158 (Ogawa) or PIC018 (Inaba) V. cholerae as described previously (28, 62). Heat-inactivated 2-fold serial dilutions of serum were incubated with guinea pig complement (Sigma) and the target strain and then allowed to grow in brain heart infusion (BHI) medium in a 96-well plate. The serum dilution that caused more than 50% reduction in target strain OD_595_ compared to saline negative-control wells was recorded as the antibody titer. A mouse monoclonal antibody, 432A.1G8.G1.H12 (28), targeting V. cholerae O1 OSP was a positive control for the assay. The limit of detection represents the lowest serum dilution at which no inhibition of growth could be detected. The *r*^2^ value was determined using a linear regression model in Prism 9 (GraphPad), and the data were represented on a log_10_ scale for ease of interpretation.

### Barcode analysis of population bottlenecks.

To create a donor plasmid containing ∼60,000 barcodes, a fragment containing the Tn*7* transposon and necessary conjugation/replication machinery from pJMP1339 (63) was generated. In addition, we amplified a kanamycin resistance cassette from pDS132-STAMPR (41) with primers containing 25 “N” nucleotides, representing ∼1 × 10^15^ possible barcodes. These fragments were assembled with the NEB HiFi DNA master mix to create pSM1. The assembly was then electroporated at scale into MFDλpir, and ∼60,000 colonies were pooled and frozen into several aliquots.

To introduce barcodes into recipient V. cholerae strains, 30 μL of thawed donor culture (MFDλpir with pSM1) was inoculated in 3 mL of LB Kan50, Carb50, and Diaminopimelic Acid (DAP) 300 μg/mL, grown overnight, and mixed with overnight culture of recipient V. cholerae strains at equal ratios (total volume of 1.2 mL) along with the donor containing the helper transposase (MFDλpir and pJMP1039). Cells were pelleted, resuspended in 100 μL of LB, and spotted on a 0.4-μm HAWP filter (Millipore). This procedure was scaled 6× to create the final library. After incubation for 4 hours at 37°C, cells were washed from the filter and plated on LB Kan50 agar. The absence of DAP counterselects against the donor strain. The resulting transconjugant colonies were then pooled and frozen and used for future infant mouse inoculations.

STAMPR library preparation was performed as previously described (41). Briefly, 2 μL of bacterial suspension was diluted in 100 μL of water and boiled for 15 min at 95°C. PCR to amplify the barcode region was performed in 2 μL of boiled cells using Phusion DNA polymerase (New England Biolabs). The presence of the correct PCR product was verified on an agarose gel, and samples were pooled, purified, and sequenced on a MiSeq (Illumina) for 78 cycles. Sequence analysis was performed using the CLC Genomics Workbench (Qiagen). To create the reference list of barcodes, we first sequenced the MFD donor library as done previously (41). After trimming the reads, sequences were deduplicated in Geneious (Biomatters) using the dedupe plugin with default settings. This resulted in a preliminary list of ∼100,000 barcodes (reference list RL1). We then deep sequenced the V. cholerae transconjugants and mapped the reads onto RL1. Consensus sequences for barcodes with at least one read were obtained to create RL2. The mapping procedure was repeated, and consensus sequences for barcodes containing more than one read were collected, creating RL3. RL3 was deduplicated in Geneious as above to create RL4. Reads were then mapped again to RL4, and consensus sequences for barcodes containing more than 1 read were obtained to create the final reference list containing 63,209 barcodes.

Reads from all samples were mapped to this reference list of barcodes and exported as a table of read counts. From these counts, we calculated Ns as done previously (41), with several modifications to bolster the initial correction for sequencing noise. Since the number of barcodes (∼60,000) is close to the sequencing depth (∼100,000 to 200,000), many barcodes have only one read mapping to them. We define *m* as the number of barcodes with greater than 1 read mapping and *n* as the number of barcodes with only 1 read mapping. The *m*/*n* ratio is used to define a noise correction factor proportional to the sequencing depth (ss). A noise correction factor, *C*, is then defined as

$$C=s_{s}(10^{\frac{m}{n}})\text{ if }\frac{m}{n}\;<\;10$$

$$C=s_{s}(10^{-\sqrt{\frac{m}{n}}})\text{ if }\frac{m}{n}\;>\;1$$

We then sampled the distribution of the entire sequencing run (which represents the noise distribution that arises by index hopping) via multinomial resampling *C* times and subtracted these reads from the output samples. One read was subtracted from all barcodes if *m*/*n* was <0.83 after this initial noise correction. In practice, these corrections remove reads from samples that are overrepresented by barcodes with only one read mapping to them, as these are likely to be noise. The remaining noise is identified by local minima in the resiliency algorithm (41).

In addition, we also used the read depth of the output sample to simulate a bottleneck on the input prior to Ns determination. This correction adjusts for the case when more barcodes would have been detected had the sample been sequenced more deeply. The full code used to analyze these data is provided in https://github.com/hullahalli/stampr_rtisan.

### Statistical analysis.

Statistical analyses were performed with Prism 9 (GraphPad). Specific statistical tests used for analysis are defined in the figure legends.

### Animal use statement.

All experiments in this study were performed as approved by the Brigham and Women’s Hospital IACUC (protocol 2016N000416) and in compliance with the Guide for the Care and Use of Laboratory Animals.
