# Supplementary figures and images for: Nontoxigenic Vibrio cholerae Challenge Strains for Evaluating Vaccine Efficacy and Inferring Mechanisms of Protection
Source: mBio. 2022 Apr 7;13(2):e00539-22. doi: 10.1128/mbio.00539-22 (PMC9040834; doi:10.1128/mbio.00539-22)

Figure S1

a

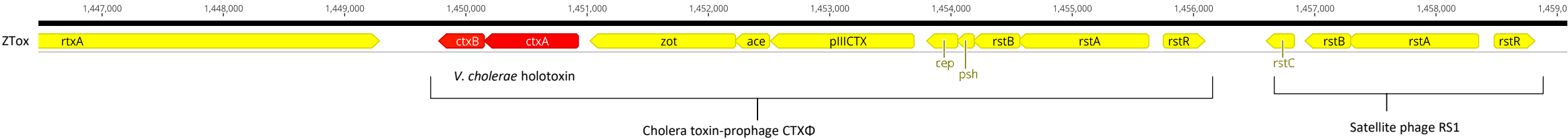

b

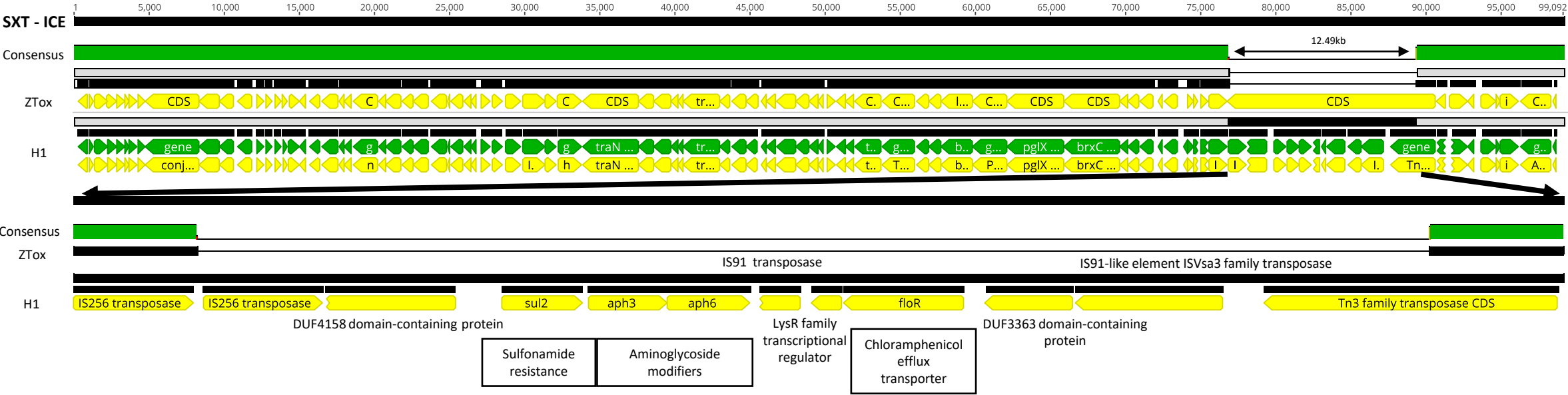

Supplement: FIG S1 [file mbio.00539-22-sf001.pdf]

Figure S2

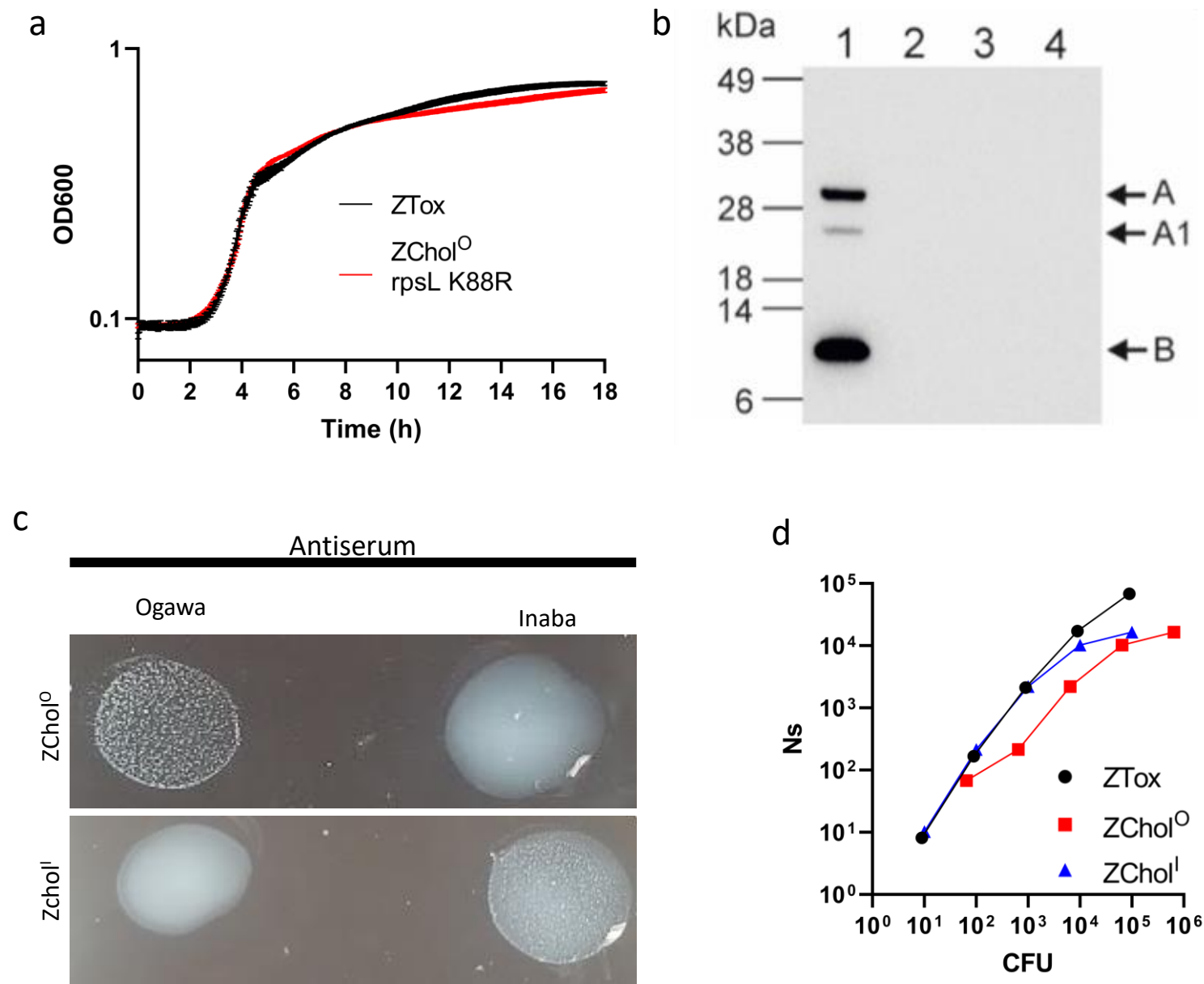

Supplement: FIG S2 [file mbio.00539-22-sf002.pdf]
