# Supplementary material for: Nontoxigenic Vibrio cholerae Challenge Strains for Evaluating Vaccine Efficacy and Inferring Mechanisms of Protection
Source: mBio. 2022 Apr 7;13(2):e00539-22. doi: 10.1128/mbio.00539-22 (PMC9040834; doi:10.1128/mbio.00539-22)
Supplement: TABLE S2 [file mbio.00539-22-st002.pdf]

ZTox.fasta

Name	Description	Contig	Location
VC0817	Putative transposae vpiT	1	Chromosome I
VC0818	Uncharacterized protein	1	Chromosome I
VC0819	Aldehyde dehydrogenase aldA	1	Chromosome I
VC0820	ToxR-activated gene A lipoprotein tagA	1	Chromosome I
VC0821	Uncharacterized protein	1	Chromosome I
VC0823	Uncharacterized protein	1	Chromosome I
VC0824	Thiol peroxidase tpx	1	Chromosome I
VC0825	Toxin coregulated pilus biosynthesis protein I tcpI	1	Chromosome I
VC0826	Toxin coregulated pilus biosynthesis protein P tcpP	1	Chromosome I
VC0827	Toxin coregulated pilus biosynthesis protein H tcpH	1	Chromosome I
VC0828	Toxin coregulated pilin tcpA	1	Chromosome I
VC0829	Toxin coregulated pilus biosynthesis protein B tcpB	1	Chromosome I
VC0830	Toxin coregulated pilus biosynthesis protein Q tcpQ	1	Chromosome I
VC0831	Toxin coregulated pilus biosynthesis outer membrane protein C tcpC	1	Chromosome I
VC0832	Toxin coregulated pilus biosynthesis protein R tcpR	1	Chromosome I
VC0833	Toxin coregulated pilus biosynthesis protein D tcpD	1	Chromosome I
VC0834	Toxin coregulated pilus biosynthesis protein S tcpS	1	Chromosome I
VC0835	Toxin coregulated pilus biosynthesis protein T tcpT	1	Chromosome I
VC0836	Toxin coregulated pilus biosynthesis protein E tcpE	1	Chromosome I
VC0837	Toxin coregulated pilus biosynthesis protein F tcpF	1	Chromosome I
VC0838	TCP pilus virulence regulatory protein toxT tcpN	1	Chromosome I
VC0839	Prepilin leader peptidase/N-methyltransferase tcpJ	1	Chromosome I
VC0840	Accessory colonization factor AcfB acfB	1	Chromosome I
VC0841	Accessory colonization factor AcfC acfC	1	Chromosome I
VC0842	RDD domain-containing protein	1	Chromosome I
VC0843	TagE protein tagE	1	Chromosome I
VC0844	Accessory colonization factor AcfA acfA	1	Chromosome I
VC0845	Accessory colonization factor AcfD acfD	1	Chromosome I
VC0847	Integrase intV	1	Chromosome I

Supp Table 2

Identity	Start	End
100	2213339	2212362
100	2211656	2212336
100	2209794	2211314
100	2209614	2206573
99.9	2206460	2204763
100	2201862	2200924
100	2200814	2200320
100	2198182	2200044
100	2197740	2197075
100	2197090	2196680
99.9	2196136	2195462
100	2195382	2194090
100	2194090	2193638
100	2193635	2192166
100	2192173	2191718
100	2191730	2190894
100	2190878	2190420
100	2190430	2188919
100	2188944	2187922
100	2187912	2186896
100	2186689	2185859
100	2185859	2185098
100	2185091	2183211
99.7	2183205	2182436
100	2182420	2181947
100	2181938	2181030
100	2180380	2181027
100	2180206	2175644
100	2172460	2173728

ZTox.fasta

Name	Description	Contig	Location
VC1758	Prophage integrase IntA	1	Chromosome I
VC1759	Uncharacterized protein	1	Chromosome I
VC1759	Uncharacterized protein	1	Chromosome I
VC1760	Putative helicase	1	Chromosome I
VC1761	VC1761	1	Chromosome I
VC1762	EH Signature domain-containing protein	1	Chromosome I
VC1763	Chemotaxis protein MotB-related protein	1	Chromosome I
VC1764	Uncharacterized protein	1	Chromosome I
VC1765	Type I site-specific deoxyribonuclease	1	Chromosome I
VC1766	Uncharacterized protein	1	Chromosome I
VC1767	DUF262 domain-containing protein	1	Chromosome I
VC1768	Methylase S domain-containing protein	1	Chromosome I
VC1769	Site-specific DNA-methyltransferase	1	Chromosome I
VC1770	Uncharacterized protein	1	Chromosome I
VC1771	Uncharacterized protein	1	Chromosome I
VC1772	WYL domain-containing protein	1	Chromosome I
VC1773	Kelch domain-containing protein	1	Chromosome I
VC1774	N-acetylneuraminate epimerase nanM	1	Chromosome I
VC1775	Uncharacterized protein	1	Chromosome I
VC1776	Putative N-acetylneuraminate lyase	1	Chromosome I
VC1777	Sialic acid TRAP transporter large permease protein SiaM	1	Chromosome I
VC1778	Sialic acid TRAP transporter small permease protein SiaQ	1	Chromosome I
VC1779	Sialic acid-binding periplasmic protein SiaP	1	Chromosome I
VC1781	Putative N-acetylmannosamine-6-phosphate 2-epimerase nanE	1	Chromosome I
VC1782	N-acetylmannosamine kinase nanK	1	Chromosome I
VC1783	N-acetylglucosamine-6-phosphate deacetylase	1	Chromosome I
VC1784	Sialidase nanH	1	Chromosome I
VC1785	Transcriptional regulator	1	Chromosome I
VC1786	UPF0758 protein	1	Chromosome I
VC1787	Uncharacterized protein	1	Chromosome I
VC1788	Uncharacterized protein	1	Chromosome I
VC1788	Uncharacterized protein	1	Chromosome I
VC1788	Uncharacterized protein	2	Chromosome II
VC1789	Transposase OrfAB, subunit B	1	Chromosome I
VC1789	Transposase OrfAB, subunit B	1	Chromosome I
VC1789	Transposase OrfAB, subunit B	1	Chromosome I
VC1789	Transposase OrfAB, subunit B	1	Chromosome I
VC1789	Transposase OrfAB, subunit B	2	Chromosome II
VC1789	Transposase OrfAB, subunit B	2	Chromosome II
VC1789	Transposase OrfAB, subunit B	2	Chromosome II
VC1791	Uncharacterized protein	1	Chromosome I
VC1791	Uncharacterized protein	1	Chromosome I
VC1792	Uncharacterized protein	1	Chromosome I
VC1792	Uncharacterized protein	1	Chromosome I
VC1793	Uncharacterized protein	1	Chromosome I

VC1793	Uncharacterized protein	1	Chromosome I
VC1794	Uncharacterized protein	1	Chromosome I
VC1794	Uncharacterized protein	1	Chromosome I
VC1795	Putative transcriptional regulator	1	Chromosome I
VC1795	Putative transcriptional regulator	1	Chromosome I
VC1796	Middle operon regulator-related protein	1	Chromosome I
VC1796	Middle operon regulator-related protein	1	Chromosome I
VC1797	Uncharacterized protein	1	Chromosome I
VC1797	Uncharacterized protein	1	Chromosome I
VC1798	Eha protein	1	Chromosome I
VC1798	Eha protein	1	Chromosome I
VC1799	Integrase catalytic domain-containing protein	1	Chromosome I
VC1799	Integrase catalytic domain-containing protein	1	Chromosome I
VC1800	Uncharacterized protein	1	Chromosome I
VC1800	Uncharacterized protein	1	Chromosome I
VC1801	Uncharacterized protein	1	Chromosome I
VC1801	Uncharacterized protein	1	Chromosome I
VC1802	Uncharacterized protein	1	Chromosome I
VC1802	Uncharacterized protein	1	Chromosome I
VC1804	Uncharacterized protein	1	Chromosome I
VC1805	Uncharacterized protein	1	Chromosome I
VC1806	G domain-containing protein	1	Chromosome I
VC1807	VC1807	1	Chromosome I
VC1808	Uncharacterized protein	1	Chromosome I
VC1809	Putative transcriptional regulator	1	Chromosome I

Identity	Start	End
100	1778425	1779660
100	1779756	1780211
89.3	2173279	2173689
100	1783140	1780342
100	1783791	1783187
100	1785233	1783782
100	1785964	1785230
100	1788077	1785957
100	1791181	1788122
100	1793209	1791218
100	1794614	1793202
100	1795943	1794615
100	1798341	1795960
100	1800545	1798482
100	1804144	1800527
100	1804217	1805077
100	1806212	1805142
100	1807381	1806227
100	1808399	1807563
100	1809424	1808528
100	1810745	1809462
100	1811262	1810753
100	1812290	1811325
100	1812517	1813227
100	1813215	1814078
100	1814082	1815218
100	1815642	1817987
100	1818310	1818134
100	1818454	1818930
100	1818927	1819064
100	1140446	1141141
100	1819855	1819160
100	2015	1320
99.8	1139272	1140449
99.8	1463891	1465065
99.8	1821029	1819852
99.8	2629815	2630992
99.8	3189	2012
99.8	357251	356074
99.8	55756	56933
100	1138270	1139229
100	1822031	1821072
100	1137695	1138054
100	1822606	1822247
100	1137275	1137646

100	1823026	1822655
100	1136636	1137214
100	1823665	1823087
100	1136297	1136617
100	1824004	1823684
100	1135936	1136310
100	1824365	1823991
100	1134665	1135126
100	1825636	1825175
100	1133553	1134662
100	1826748	1825639
100	1131824	1133581
100	1828477	1826720
100	1130846	1131817
100	1829455	1828484
100	1130172	1130507
100	1830129	1829794
100	1129946	1130182
100	1830355	1830119
100	1129117	1128854
100	1128803	1128357
100	1128250	1127264
100	1126589	1127229
100	1126297	1125452
100	1125137	1125337

ZTox.fasta

Name	Description	Contig	Location	Identity	Start
VC0174	SPOR domain-containing protein	1	Chromosome I	100	2995980
VC0175	Deoxycytidylate deaminase-related protein	1	Chromosome I	100	2993191
VC0176	Putative transcriptional regulator vspR	1	Chromosome I	100	2992365
VC0177	transcriptional regulator vspR	1	Chromosome I	100	2992262
VC0178	Patatin-related protein capV	1	Chromosome I	100	2990785
VC0179	Cyclic GMP-AMP synthase dncV	1	Chromosome I	100	2989698
VC0180	ThiF domain-containing protein	1	Chromosome I	99.9	2988391
VC0181	Prok-JAB domain-containing protein	1	Chromosome I	100	2986644
VC0182	Uncharacterized	1	Chromosome I	100	2985735
VC0183	Phage integrase int	1	Chromosome I	100	2983652
VC0184	Uncharacterized	1	Chromosome I	100	2981954
VC0185	Putative transposase	1	Chromosome I	100	2980755
VC0186	Glutathione reductage gorA	1	Chromosome I	100	2979314
VC RS0086	Uncharacterized	1	Chromosome I	100	2992882
VC RS0087	Uncharacterized	1	Chromosome I	100	2991282

End
2995033
2994780
2992673
2991699
2989718
2988394
2986637
2986177
2986166
2985763
2983642
2981957
2980666
2993115
2991034

ZTox.fasta

Name	Description	Contig	Location	Identity	Start	End
VC0489	Putative hemolysin	1	Chromosome I	100	2637735	2639495
VC0490	Uncharacterized protein	1	Chromosome I	100	2635012	2636973
VC0491	Uncharacterized protein	1	Chromosome I	100	2634475	2635011
VC0492	Uncharacterized protein	1	Chromosome I	100	2633340	2634506
VC0493	Uncharacterized protein	1	Chromosome I	100	2632209	2633084
VC0494	Uncharacterized protein	1	Chromosome I	100	2631824	2631180
VC0500a	TransposaseOrfAB,subunit a	1	Chromosome I	82.5	1139447	1139583
VC0500a	TransposaseOrfAB,subunit a	1	Chromosome I	82.5	1464063	1464199
VC0500a	TransposaseOrfAB,subunit a	1	Chromosome I	82.5	1820854	1820718
VC0500a	TransposaseOrfAB,subunit a	1	Chromosome I	82.5	2629990	2630126
VC0500a	TransposaseOrfAB,subunit a	2	Chromosome II	82.5	3014	2878
VC0500a	TransposaseOrfAB,subunit a	2	Chromosome II	82.5	357076	356940
VC0500a	TransposaseOrfAB,subunit a	2	Chromosome II	82.5	55931	56067
VC0501b	Transposase	1	Chromosome I	71.3	2211656	2212115
VC0513	AraC family transcriptional regulator	1	Chromosome I	99.9	2628886	2628071
VC0514	Methyl-accepting chemotaxis protein	1	Chromosome I	100	2628074	2626194
VC0515	Putative signal transduction protein	1	Chromosome I	100	2626090	2624858
VC0516	Phage integrase	1	Chromosome I	100	2623227	2624468
VC0517	RNA polymerase sigma factor RpoD	1	Chromosome I	100	2620964	2622841
