# Supplementary material for: Nontoxigenic Vibrio cholerae Challenge Strains for Evaluating Vaccine Efficacy and Inferring Mechanisms of Protection
Source: mBio. 2022 Apr 7;13(2):e00539-22. doi: 10.1128/mbio.00539-22 (PMC9040834; doi:10.1128/mbio.00539-22)
Supplement: TABLE S1 [file mbio.00539-22-st001.pdf]

**Antibiotic**	**ZChol^{O}**		**ZChol^{I}**	
	MIC (µg/mL)	Interpretation	MIC (µg/mL)	Interpretation
Ampicillin	4	S	4	S
Azithromycin	0.5	S	0.5	S
Ciprofloxacin	0.5	S	0.5	S
Erythromycin	2	N	2	N
Tetracycline	1	S	1	S
Sulfamethoxazole/Trimethoprim	0.25	S	0.25	S
Streptomycin	>200	R	>200	R

Supp Table 1
